# Cyclic vomiting syndrome: Future clinical and research priorities

**DOI:** 10.1111/nmo.14825

**Published:** 2024-05-22

**Authors:** William L. Hasler, B. U. K. Li, David J. Levinthal, Thangam Venkatesan

**Affiliations:** 1 Mayo Clinic Arizona, Scottsdale, Arizona, USA; 2 Medical College of Wisconsin, Milwaukee, Wisconsin, USA; 3 University of Pittsburgh, Pittsburgh, Pennsylvania, USA; 4 Ohio State University, Columbus, Ohio, USA

**Keywords:** antiemetic agents, cannabinoid hyperemesis syndrome, epidemiology, pathophysiology, prophylactic therapy

## Abstract

**Background:**

An increasing number of studies have explored the clinical features, epidemiology, pathophysiology, and management of cyclic vomiting syndrome (CVS). CVS is common in adults and children and negatively impacts patients, families, and the healthcare system. A related condition, cannabinoid hyperemesis syndrome (CHS), has been a focus of interest in the lay press and published literature.

**Purpose:**

Clinical presentations of CVS have been defined by small series and expert opinion, but recent prospective studies are refining our understanding of the spectrum of emetic episodes and the breadth of comorbid conditions. Large cross-sectional population analyses are clarifying CVS prevalence and factors related to age, ethnicity, and geographic region. CVS pathophysiology is multifactorial with contributions from migraines, dysautonomia, endogenous cannabinoids, mitochondrial dysfunction, genetic abnormalities, and rapid gastric emptying. CVS treatment relies on antiemetics and antimigraine therapies to abort acute episodes coupled with prophylactic regimens employing neuromodulators and antiepileptics. CHS represents a challenge partly because of difficulties in achieving sustained cannabis abstinence. Benefits of other therapies in CHS remain poorly defined. Several areas warrant further scrutiny including better identification of CVS triggers and characterization of different CVS subsets including those with frequent severe episodes, refined description of epidemiology to allow targeting of populations predisposed to CVS development, rigorous definition of pathogenic factors to provide a foundation for exploratory studies of novel therapies, and conduct of controlled trials by multicenter collaborations to confirm benefits of existing and new therapies in development. Progress in these areas will be facilitated by generous governmental and industry support.

## 1 | INTRODUCTION

Research relating to clinical phenotypes, epidemiology, pathophysiology, and management of cyclic vomiting syndrome (CVS) has burgeoned in recent decades. Whereas CVS was once considered only a pediatric disorder, it is now known to affect 2% of adults in the United States,^1^ which is similar to rates in children.^2,3^ Several smaller landmark studies provided foundational understanding of the clinical characteristics of CVS. Recent larger single-center studies, employing cross-sectional analyses of large databases, are providing detailed information on CVS distribution by gender, age, ethnicity, and other clinical characteristics.^4–6^ Study of neurologic and other conditions comorbid with CVS has provided insight into its potential underlying causes. Other factors including autonomic nervous system dysregulation, endocrine and endocannabinoid disturbances, mitochondrial dysfunction, and genetic abnormalities have recently been proposed to have pathogenic roles in subsets of CVS.^7–10^ Most research on treatment of CVS comes from uncontrolled open label trials, and retrospective studies from single centers.^11,12^ In 2019, comprehensive adult guidelines based on GRADE methodology rather than expert consensus laid the foundation for standardizing management of CVS based on evidence.^13^

A related clinical condition, cannabinoid hyperemesis syndrome (CHS), presents with similar episodes of relentless vomiting in patients who use of large quantities of cannabis for a prolonged time period.^14^ CHS has dominated both the lay press and literature reports of rising numbers of cases over the past 15–20 years.^15,16^ It is a matter of debate whether CHS is a distinct entity or represents a subset of CVS. The mechanisms for initiation of CHS remain obscure and information on successful therapies of this disorder other than cannabis abstinence is limited.

Despite these advances, much additional investigation is required to be able to offer optimal evaluation and therapy of CVS and CHS. Further progress will be made with involvement of the federal agencies like the National Institutes of Health and the pharmaceutical industry to provide both financial support and the multicenter infrastructure needed to conduct controlled studies to define effective acute and preventive therapies for well-defined CVS subsets. To date, funding for CVS investigation has been limited with only 16 trials (two recruiting, 11 completed, three withdrawn or terminated early) currently listed on clinicaltrials.gov. Of these, only two have been funded by industry and none by federal sources.

## 2 | CLINICAL FEATURES OF CVS

### 2.1 | Established data

Diagnostic criteria for CVS were defined by the Rome Foundation for adults in 2006 and then revised in 2016 and for children by the North American Society for Pediatric Gastroenterology, Hepatology and Nutrition in 2008.^17–19^ These criteria are mostly qualitative but emphasize the main characteristics of repeated severe episodic vomiting episodes with intervening periods with substantially reduced symptom intensity. A CVS diagnosis is commonly made based on the typical clinical presentation of episodic nausea and vomiting in the absence of other potential etiologies. While nausea and vomiting are cardinal symptoms of CVS. Abdominal pain is noted by almost 90% of patients, can manifest during the prodromal phase, and has been reported to occur after onset of nausea in one study.^6^ Many autonomic symptoms are also seen during an episode including pallor, hypersalivation, diaphoresis, hot flashes, and postural changes in pulse and blood pressure. In one cross-sectional study, autonomic symptoms were reported by 92% of CVS patients.^20^ Triggers of CVS episodes are inconsistently identified, however, increases in stress (including “good” stressors like holidays and “bad” stresses from conflicts) and sleep disturbance are often reported. Recognition of the heterogeneity of presentation by clinicians is crucial. Only limited evaluation is necessary to make a CVS diagnosis, as abnormal findings on laboratory, brain imaging, and gastrointestinal endoscopy are found in <5% of patients. The differential diagnosis of CVS includes other episodic conditions including migraines, epilepsy, brain masses, hepatic porphyria, mitochondrial dysfunction, and other neurologic disorders, though these are uncommon and can be excluded with a careful history and targeted investigations.^20^

Recent research provides more detail on profiles of CVS episodes. Robust quantitative data on CVS episode characteristics were provided by a preliminary study of the natural history of the disorder in adults.^21^ In this study, 61/88 (69.3%) reported at least 1 CVS episode over 6 months of observation, with a mean episode duration of 7.1 ± 12.0 days. However, 26.7% of episodes were >7 days long exceeding the limit proposed by the Rome criteria.^18^

**FIGURE 1:**
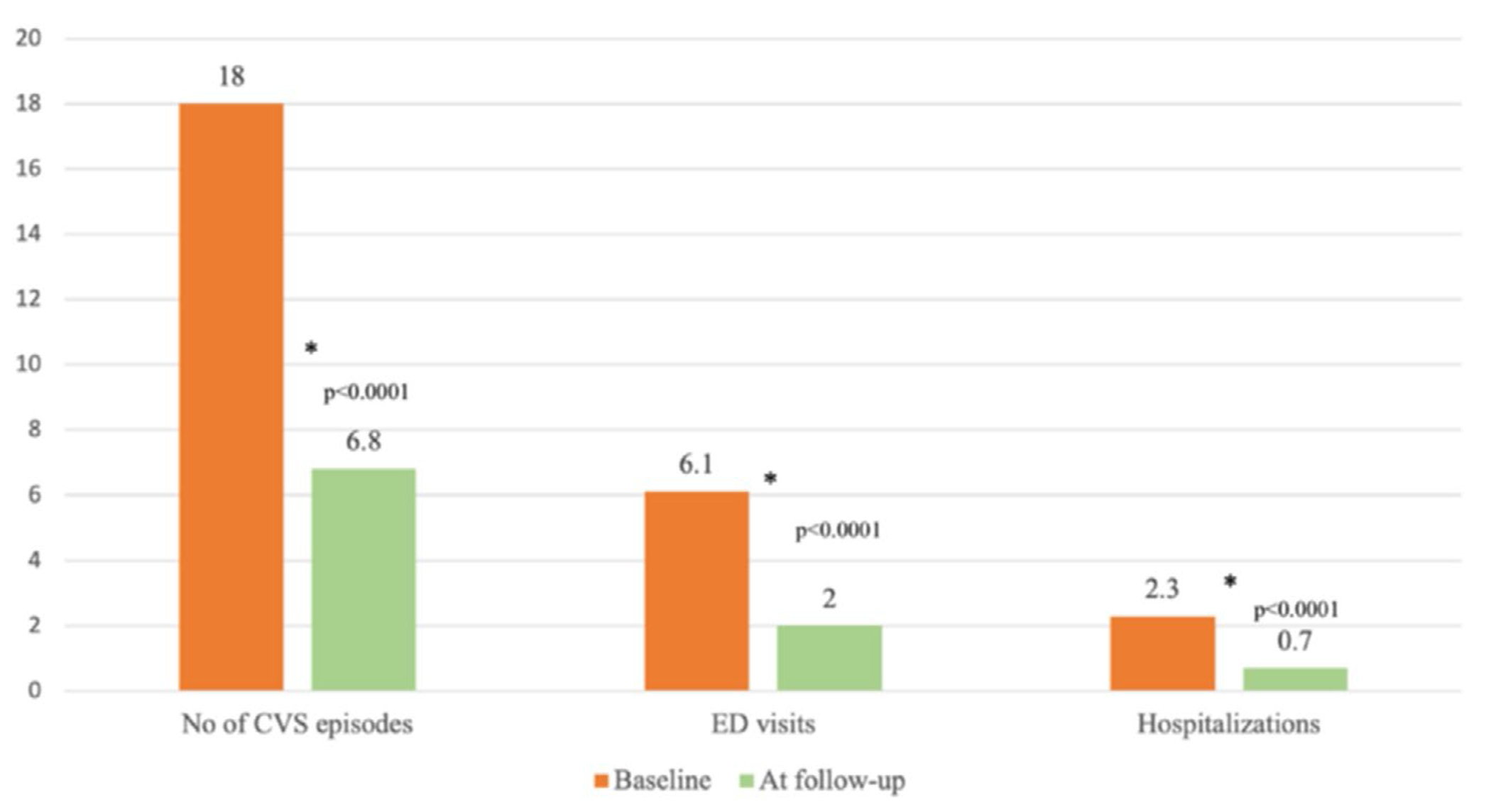
Changes in CVS episode frequency, emergency department (ED) visits, and inpatient hospitalizations are shown for CVS patients in a tertiary center. All three parameters decreased significantly compared to baseline over a mean of 47 months. From reference 4.

There is limited understanding of the long-term outcomes of adults with CVS. In retrospective analyses from a tertiary center over a mean of 47 months, annual CVS frequency in treated patients decreased from 18 to 6.8 episodes, ED visits were reduced from 6.1 to 2, and hospitalizations decreased from 2.3 to 0.7 (Figure 1).^4^ Although many CVS patients use cannabis products for symptom relief, chronic cannabis use was associated with reduced odds of CVS resolution in this study. In contrast, 40% of a group of 100 children with CVS progressed to chronic autonomic nervous system dysfunction at a mean age of 15 years.^22^

### 2.2 | Priorities for future investigation

Further details on features of CVS in adults and children will be possible by generation of large longitudinal multicenter patient databases using standardized and validated questionnaires. This will enhance our understanding of symptom profiles, episode characteristics, comorbid conditions, and natural history in different subsets of patients (Table 1). As part of this initiative, consistent use of the existing ICD 10 code for cyclical vomiting syndrome unrelated to migraine (R11.15) should be encouraged when appropriate.

## 3 | EPIDEMIOLOGY OF CVS

### 3.1 | Established data

**FIGURE 2:**
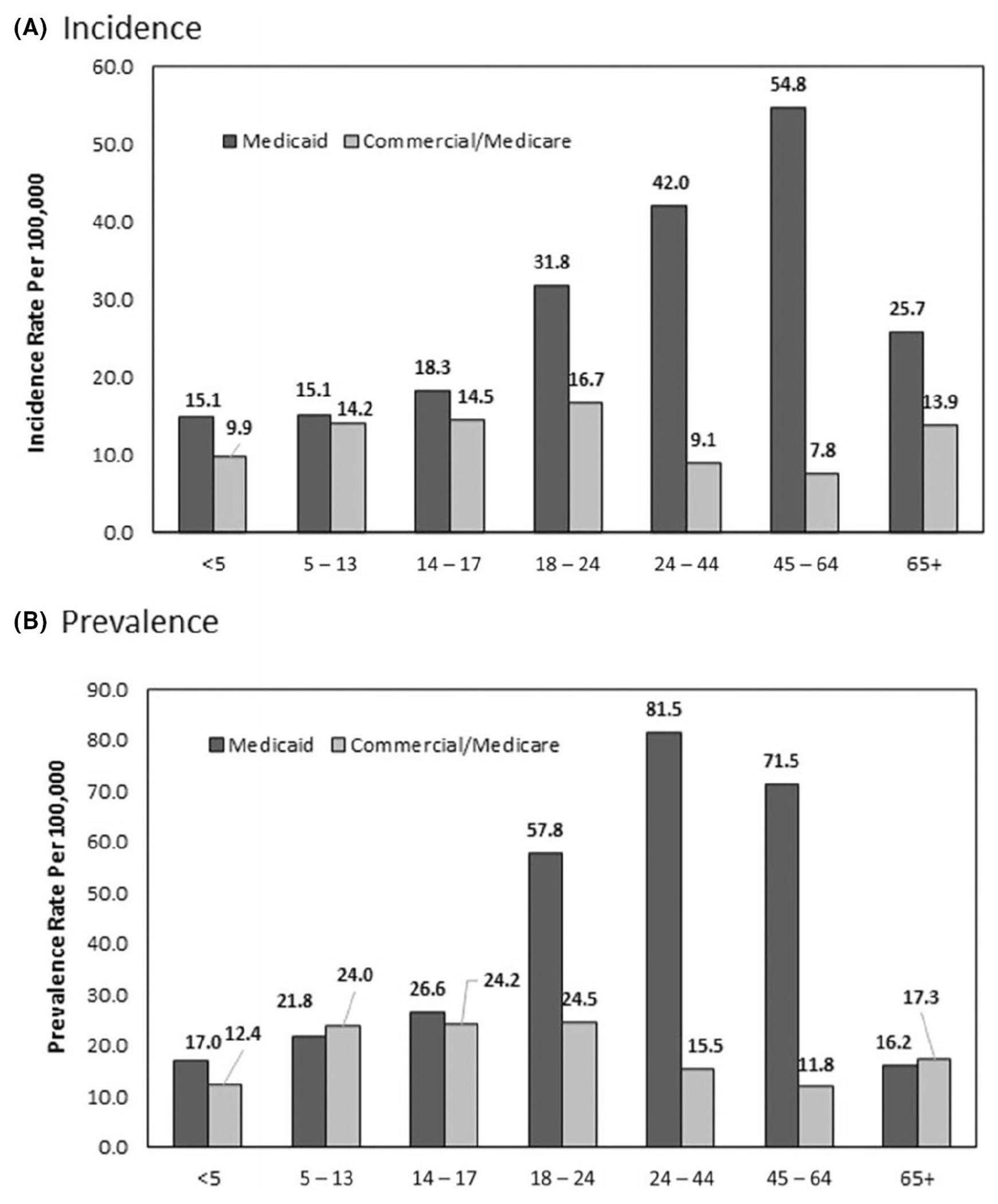
Incidence and prevalence data for CVS is shown for a large cross-sectional study of claims from Commercial/Medicare and Medicaid database as a function of patient age. From reference 24.

Epidemiology of CVS has been a topic of extensive investigation. Among 920 patients referred from primary to secondary outpatient care in a UK gastroenterology clinic health system, 10.8% were judged to have CVS based on satisfaction of Rome III criteria for CVS in the absence of other “organic” diagnoses.^23^ However, many patients in this cohort also fulfilled criteria for other functional esophageal (functional heartburn and functional chest pain), gastroduodenal (functional nausea, functional belching, and postprandial distress syndrome), and bowel (irritable bowel syndrome) disorders. Insight on CVS epidemiology has been provided by newer population-based reports. A cross-sectional study of Marketscan^®^ claims data comprised of more than 15 million persons observed a prevalence of CVS of 16.7 per 100,000 individuals in a large Commercial/Medicare database and 42.9 per 100,000 individuals from a Medicaid database.^24^ In this same report, CVS incidence was 10.6 and 26.6 per 100,000 individuals from these two databases, respectively. Peak prevalence was noted from 5 to 24 years of age in the Commercial/Medicare database and 18 to 64 years of age in the Medicaid database (Figure 2). Highest prevalence rates were found in the US Midwest while the lowest rates were in the Northeast. CVS was more common in White/Caucasian persons compared to Black/African American and Hispanic populations. Initial CVS diagnosis in adults was made as an inpatient in 37.2% of the Commercial/Medicare database and 42.3% of the Medicaid population. The authors of that article speculated that the higher rates in the Medicaid database stemmed from socioeconomic factors that are known to increase susceptibility to other chronic diseases compared to the general population. Furthermore, they proposed that the relatively higher CVS prevalence in older populations in the Medicaid cohort likely was a result of barriers to access for younger individuals due to unemployment or disability. Causes of the higher apparent prevalence in the Midwest are unknown, although specialists in CVS care are concentrated in that geographic region suggesting a possible role for greater recognition of the syndrome. Another report identified risk factors for inpatient hospitalization of CVS patients including non-Caucasian race, lack of insurance coverage, and public health insurance coverage.^25^ Conditions comorbid with CVS included anxiety (32%–39%), depression (26%–34%), cardiac conditions (39%–42%), and acid reflux (30%–40%).

Most research on CVS epidemiology has focused on US populations, likely due to more broad-based adoption of accepted diagnostic criteria and increased awareness of the syndrome. For example, an earlier Rome Foundation survey of chronic disorders of nausea and vomiting queried patients only in the United States, UK, and Canada.^1^ Prevalence and incidence data are only now being generated for other regions of the world. A newer cross-sectional database study from Japan observed a CVS prevalence in adults of five per 100,000 population, a much lower rate than seen in the United States, although prevalence in children was much higher at 210 per 100,000 in this report.^26^

### 3.2 | Priorities for future investigation

Gleaning more detailed information on CVS epidemiology will require access to comprehensive international databases comprised of diverse patient subsets in relation to age, ethnicity, geographic region, comorbid illness, and other factors (Table 1). Such registries have already been generated for other disorders of chronic nausea and vomiting-like gastroparesis with generous federal support. Recent reports indicate that CVS is likely more prevalent than gastroparesis and similar in prevalence to pediatric inflammatory bowel disease. Much of CVS epidemiology can be defined using insurer population databases, but these populations rely on coding protocols which exhibit problems relating to assigning the correct diagnosis to enrollees and relying only on inpatient, emergency department (ED), or outpatient resource utilization as sources for their data. One goal of future study should be to better understand factors that contribute to greater CVS prevalence and incidence in different ethnic or socioeconomic groups. Furthermore, they often exclude patients not covered by health insurance. Recruitment of financial and structural support for CVS epidemiology investigation from governmental and industry sources offers the potential to compensate for these limitations.

## 4 | PATHOPHYSIOLOGY OF CVS

### 4.1 | Established data

CVS appears to have a heterogeneous pathophysiology, with separate functional abnormalities playing more dominant roles in different patient subsets. Several neurologic and non-neurologic factors have been identified in association with CVS and are described below.

**FIGURE 3:**
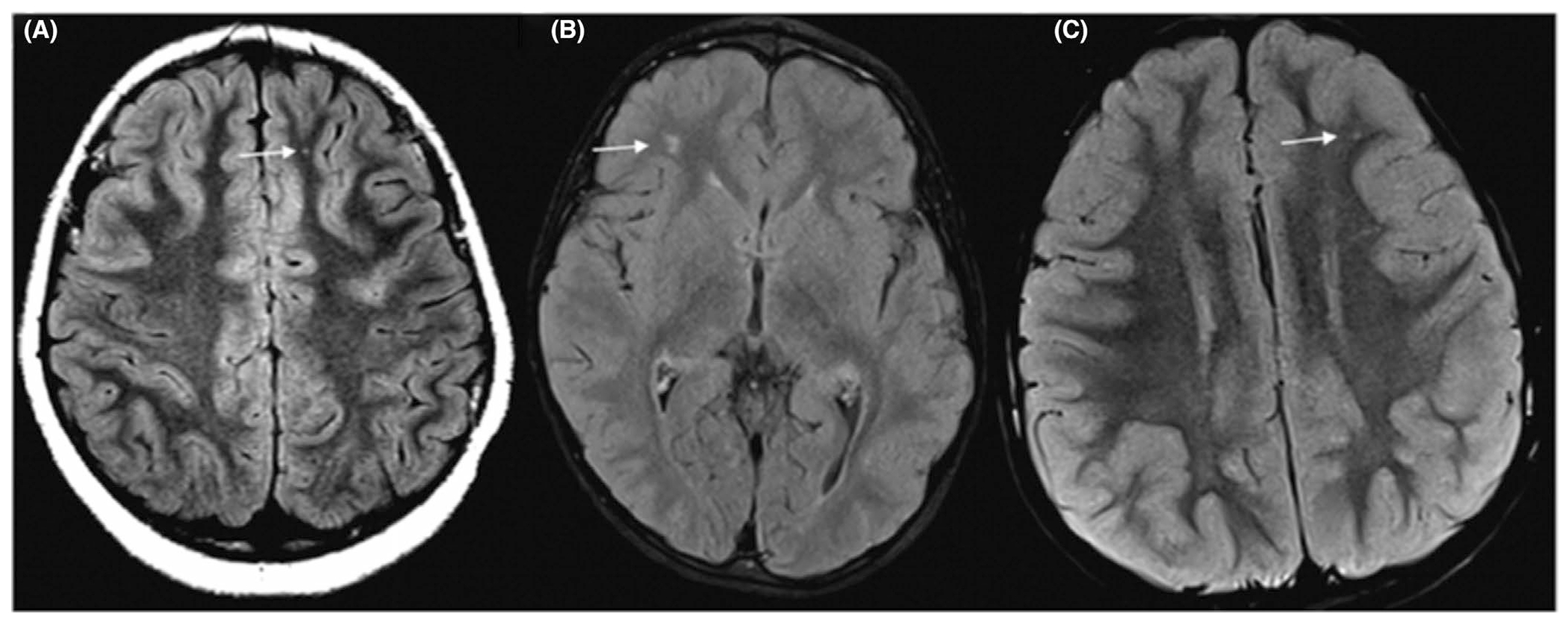
Brain MRI images using axial T2-weighted fluid-attenutated inversion recovery sequences are shown for 3 patients with CVS and migraine headaches who show evidence of hyperintensities in the subcortical white matter (arrows). From reference 27.

Migraine headache is the neurologic disorder that is most often comorbid with CVS. Different series report inconsistent association with between 6% and 78% of adult patients with CVS either having personal or family histories of migraines; this association is stronger with pediatric CVS. Both conditions are episodic, present with photophobia and symptoms as well as objective findings of dysautonomia, and respond to antimigraine therapies including triptans to abort acute symptoms and tricyclics and topiramate for prophylaxis. In one study, brain MRI scans exhibited white matter hyperintensity similar to migraines in three of 13 patients with CVS plus migraines versus none of five patients with CVS but no migraines (Figure 3).^27^ Another investigation observed similar reductions in insular connectivity with brain sensorimotor regions in migraines and CVS.^28^ There has been a resurgence in interest in the association of abdominal migraine with CVS, which also responds to antimigraine therapy.

In one report, 40% of children and 50% of adults with CVS met criteria for rapid gastric emptying as defined by <37% retention of a solid ^99m^Tc-l abeled meal at 1 h and/or <30% retention at 2 h.^29^ Rapid gastric emptying was associated with autonomic dysfunction in another study.^30^ Sympathetic autonomic nervous system dysfunction is prevalent in CVS and includes postural orthostatic tachycardia, sudomotor abnormalities, and respiratory sinus arrhythmias. Recent studies in children have demonstrated that patients with reduced vagal efficiency are more likely to respond to neuromodulation devices like the IB-stim, which is approved for treatment in pediatric disorders of gut–brain interaction (DGBI) and may improve chronic nausea, vomiting, and abdominal pain.^31,32^ The endocannabinoid system may also play a role in disease pathogenesis. Endocannabinoids N-arachidonylethanolamine (AEA) and 2-arachidonoylglycerol (2-AG), mobilized during periods of stress, did not increase during a CVS attack, raising the possibility of endocannabinoid dysfunction involvement. The same study demonstrated elevated salivary cortisol levels in cannabis users compared to non-cannabis users supporting an enhanced stress response.^7^ A separate study described differences in endocannabinoid responses and related lipids in CVS compared to controls, with higher 2-AG and 2-oleoylglycerol levels at baseline and selective increases in oleoyl ethanolamine after sham feeding in those with CVS.^33^

A case–control study showed that single nucleotide polymorphisms of genes encoding for the cannabinoid CB_1_ receptor (CNR1) were associated with higher risk of CVS. The AG and GG genotypes of CNR1 rs806380 were associated with an increased risk of CVS, whereas the CC genotype of CNR1 rs806368 was associated with a decreased risk.^34^ Genetic polymorphisms involving the μ-opioid receptor and calcium channel gene type-2 ryanodine receptor were also associated with CVS.^35^

Several additional factors have also been proposed for CVS pathogenesis. Anxiety, depression, and somatization disorders commonly affect CVS patients and CVS episodes may be associated with psychological stress and catastrophizing. In catamenial CVS, episodes present stereotypically at the times of menstruation, exhibiting a temporal association similar to menstrual migraines that suggests hormonal influences.^36^ Mitochondrial disorders have been associated mostly in pediatric cases of CVS and often exhibit a maternal inheritance pattern. Two mitochondrial DNA polymorphisms (16519T, 3010A), when combined, are more prevalent in children with CVS than in controls.^8^ In a whole exome/genome sequencing study, 12 genes were identified as highly likely associated with CVS, and 13 genes were possibly related to the disorder.^37^ The authors of that article speculated that these polymorphisms, which affect either cation transport or energy metabolism, could induce vagal hyperexcitability and autonomic dysfunction in a vicious cycle pattern.

### 4.2 | Priorities for future investigation

Although these diverse abnormalities do show associations with subsets of CVS, their roles in the causation of CVS episodes have not been proved and the mechanisms by which these factors elicit episodic vomiting are poorly understood. Future research should be dedicated to making the connections between these factors and genesis of symptoms and to further identify any previously undefined pathways that may underlie CVS episodes (Table 1). Furthermore, investigations should address how interactions between genetic and neurobiobehavioral factors affect CVS. Information gleaned from such focused studies will form the foundation for study of more targeted therapies of CVS. As with proposed investigations into CVS epidemiology, generation of patient registries of sufficient size will permit recruitment into focused mechanistic studies to be conducted by laboratories with expertise in basic and translational science.

## 5 | TREATMENT OF CVS

### 5.1 | Established data

Treatment of CVS includes two major components—abortive therapy to be administered at the onset of a CVS episode and continued for its duration, and prophylactic therapy to be given with the goal of reducing frequency and intensity of subsequent flares. Prophylactic therapy is typically considered for patients with moderate–severe CVS given the risk–benefit ratio of benefits versus side effects associated with currently available therapies. Most publications of abortive or prophylactic therapies of CVS report on their benefits in uncontrolled studies comprised of small, single-center samples.

A range of antiemetic and antimigraine medications are reported to have efficacy in reducing severity of CVS attacks. In the large cross-sectional study of Marketscan^®^ claims data described previously, only 47%–55% of patients were given acute medication treatments for CVS within 30 days of initial diagnosis including serotonin 5-HT_3_ antagonists in 38.4%, dopamine D_2_ antagonists in 18.3%, and neurokinin NK_1_ receptor antagonists in 0.6%.^24^ The 5-HT_3_ antagonist ondansetron and the NK_1_ antagonist aprepitant have shown benefit in a systematic review of ED management of pediatric CVS. One difficulty of care of CVS is that the relentless vomiting can preclude adequate absorption of oral formulations of drugs. Some agents with D_2_ antagonism can be administered as rectal suppositories in CVS including prochlorperazine and promethazine.^38^ Similarly, a recent report reported good response to rectal diazepam in two children with acute CVS flares.^39^ Because of the proposed shared pathogenesis of CVS and migraine headaches, antimigraine therapies have been employed to abort CVS episodes. Subcutaneous sumatriptan reduced vomiting frequency by >50% in 69% of children in the previously mentioned prospective trial.^38^ Another report found injectable antimigraine triptans to be effective in children with mild CVS flares lasting <24 h.^40^ In a cross-sectional study of 115 adults with CVS, intranasal sumatriptan improved symptoms of nausea and vomiting in >50% of patients within 2 h of administration.^41^

**FIGURE 4:**
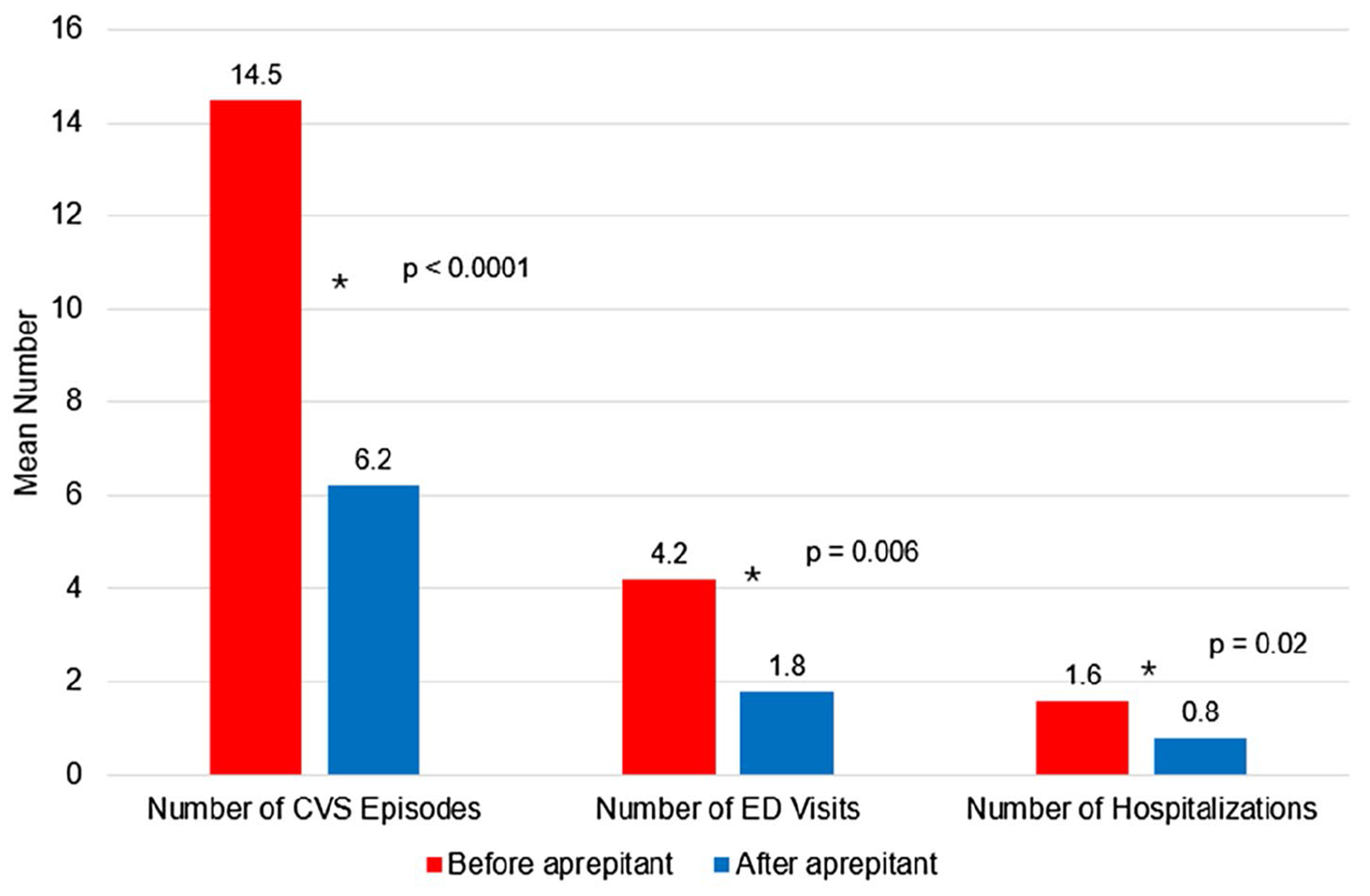
The response to aprepitant as prophylaxis for CVS is shown. Annualized numbers of CVS episodes, ED visits, and inpatient hospitalizations decreased significantly compared to the year before starting aprepitant. From reference 46.

Neuromodulators and other therapies have been proposed for CVS prophylaxis. In the aforementioned study using Marketscan^®^ claims database, only 32%–35% took medications for CVS prevention in the initial 30 days after diagnosis including tricyclic agents in 9.6% and anticonvulsants in 15.8%.^24^ Across studies, tricyclics resulted in a partial response in 413/600 (69%) of adult and pediatric CVS patients.^12^ Some have proposed comparing responses in those who are slow versus rapid tricyclic metabolizers. One such study in adult CVS patients observed that 4% exhibited poor metabolism of amitriptyline while 43% were ultrarapid metabolizers.^42^ Those who were normal CYP2D6 metabolizers showed trends to better response to amitriptyline prophylaxis than abnormal metabolizers (64% vs. 36%, *p* = 0.06). Anticonvulsants used for CVS prophylaxis include topiramate, levetiracetam, zonisamide, valproic acid, and phenobarbital. In a recent study, 65% of adult CVS patients reported reduced yearly episodes, ED visits, and hospitalizations on topiramate.^43^ Factors associated with response included higher doses, longer use, and topiramate monotherapy. Another group observed efficacy of topiramate in 85% of a pediatric cohort, and proposed that this agent should be considered for primary CVS therapy in children.^44^ Patients refractory to tricyclic agents showed 50% reductions in CVS episodes on zonisamide or levetiracetam in an older uncontrolled report in adults.^45^ Seventy-one percent of adult CVS patients improved on scheduled prophylactic regimens of the NK_1_ antagonist aprepitant (daily in 23%, three times weekly in 73%, and a few times monthly in 4%) (Figure 4).^46^ Other agents with preventative benefits in CVS include cyproheptadine, beta blockers (propranolol, nebivolol), and mitochondrial supplements (L-carnitine, coenzyme Q10).

Small randomized, controlled trials (RCTs) conducted in children in Iran reported benefits of amitriptyline In the first study of 64 pediatric CVS patients followed for 6 months, frequencies and durations of CVS attacks were similar on amitriptyline (1 mg/kg/day) and cyproheptadine (0.2 mg/kg/day).^47^ In the second investigation of 72 children with CVS followed for 3 months, amitriptyline (1 mg/kg/day) showed superior reductions in attack duration versus topiramate (1–2 mg/kg/day) and more patients stopped having attacks on amitriptyline than topiramate.^48^

Psychological therapies (behavioral modification and adaptive coping) have been advocated for CVS subsets but data supporting their use are sparse. Transcutaneous auricular vagal stimulation (tVNS) is approved for treatment of migraines and epilepsy and has been found to be effective in 55%–65% of children with refractory CVS.^49^ It has been proposed that tNVS engages the nucleus tractus solitarius with subsequent activation of the locus ceruleus, amygdala, and nucleus accumbens in the brain.^50^

Novel therapies have been proposed or are in testing for CVS. The N-methyl-D-aspartate antagonist ketamine has demonstrated the ability to abort a CVS episode in a 40-year-old CVS patient.^51^ In unpublished retrospective data on 108 ED visits in 20 CVS patients, ketamine at doses >15 mg was associated with inpatient hospitalization rates of 31% while doses <15 mg were associated with 100% hospitalization rates.^52^ It has also been used as preventative therapy in one patient.^53^ Taken together, the published data are not yet robust enough to advocate routine ketamine use for acute or chronic CVS care until more experience with this therapy is presented in peer-reviewed format. The butyrophenone D_2_ antagonist haloperidol is established for acute care of CHS, but this agent did not affect admission rates from the ED or repeat visits to the ED within 30 days in a retrospective study of 43 CVS patients.^54^ Because of the close association of migraines to CVS, some have suggested that newer classes of anti-migraine therapies may be beneficial in CVS as well. Small molecule calcitonin gene-related peptide (CGRP) receptor antagonists (gepants) and monoclonal antibodies that target CGRP (e.g. galcanezumab) show potent effects on chronic migraines. Triptans bind to 5-HT_1F_ receptors and blunt CGRP release from trigeminal nerves in patients with migraines. The role of CGRP in the induction of CVS episodes is unknown.

### 5.2 | Priorities for future investigation

Future treatment trials should strive to enroll CVS patients in multicenter, double-blind comparator studies with strict inclusion criteria and in large enough numbers to perform subgroup analyses to characterize which patients respond to a given therapy (Table 1). Older antiemetics such as ondansetron and established prophylactic neuromodulators-like tricyclic agents have already been “grandfathered” into standard clinical care of CVS. As a consequence, it is uncertain if RCTs of these therapies will be feasible in CVS although RCTs of tricyclics have been successfully conducted for functional dyspepsia and gastroparesis albeit with needs for prolonged subject recruitment to achieve the desired sample size goals. However, less commonly prescribed, newer therapies including aprepitant or selected antiepileptic agents may be more easily evaluated in structured, controlled trial settings. Given that many patients do not respond to standard abortive and preventative therapies, dedicated delineation of the benefits of novel drug classes including antimigraine treatments should be pursued. Because it has purported efficacy in pediatric CVS, tVNS should be studied comprehensively in adult populations including defining which stimulation parameters offer the best response. Securing reliable funding from federal and industry sources will be critical to accomplish these goals.

## 6 | IMPACT OF CVS

### 6.1 | Established data

CVS has a significant impact on patients, families, and the health care system partly due to underdiagnosis or misdiagnosis and partly to a lack of safe, effective, and targeted therapies supported by robust trials. Consequently, approximately 30% of patients undergo futile surgical procedures like cholecystectomy. At one single center, 35.9% of 181 adult CVS patients visited an ED a total of 228 times.^55^ Howeve r, six patients were responsible for 42% of ED visits and hospitalizations suggesting there is a small but highly impaired CVS subset requiring intensive resource use. Another web-based survey found high health care utilization with a median of 15 ED visits in adults with for CVS ranging from 1 to 200. That the diagnosis was not recognized in the ED >95% of the time highlights the need for increasing awareness about CVS in the medical community.^56^ Furthermore, there may be misconception among some providers that CVS is commonly a consequence of cannabis use. Poor patient experiences and subsequent distrust of the health care system leads patients to visit different health systems, not seek care, or to see multiple providers.^57^ It is conceivable that CVS patients may also experience significant stigmatization as a consequence of frequent ED visits. Although the impact of stigma has not been directly studied in CVS, reports in other DGBIs confirm that self-stigmatization can increase symptoms, worsen compliance with medical regimens, and construct barriers to pursuing care.^58^

More recent data using Marketscan^®^ commercial databases reinforce the high health care costs and burden. Annualized total health care costs in patients with CVS were ~4 times higher compared to non-CVS controls ($57,140 vs. $14,912) and were primarily driven by inpatient stays.^59^ As a result, patients with CVS had longer periods of short-term disability (21 vs. 7 days) and absenteeism (26 vs. 22 days) compared to matched non-CVS patients.^60^

Caregivers of CVS patients are also affected leading to further loss of productivity and, in some extreme cases, disruption of the family unit. While comorbid conditions like anxiety and depression are present in ~50% of patients, posts about mental health needs were made by 9% of patients in a recent study analyzing social media.^61^ In summary, CVS is a significant public health problem and has a significant direct and indirect burden with high health care utilization in a subset of patients. This warrants multitiered strategies to alleviate the suffering of patients with this debilitating disorder.

### 6.2 | Priorities for future investigation

Firstly, major initiatives should be taken to increase awareness of CVS, improve diagnosis and access to therapy. While the 2019 ANMS guidelines provide a foundational basis for management of CVS based on a biopsychosocial model of care, several major obstacles remain. Education of the medical community and dissemination of knowledge about CVS beyond the GI community to hospitalists, ED physicians, and primary care providers remains important. This can lead to prompt diagnosis and intervention. Using electronic reminders and artificial intelligence innovations to “flag” patients with recurrent unexplained vomiting will result in improved recognition. While a biopsychosocial model using multidisciplinary teams has been recommended, care continues to be disease-centric as opposed to an integrated model of care that can improve patient-centered outcomes.^62^ Finally, funding for research into targeted therapies for CVS are needed given the limited options now available.

## 7 | PRIORITIES FOR CHS INVESTIGATION

CHS differs from CVS by its onset after prolonged use of large quantities of cannabis and its relief by prolonged cannabis abstinence.^18^ One challenge of managing suspected CHS is obtaining accurate characterization and quantification of cannabis use in patients presenting with episodic vomiting. In one study, only 53% of patients informed primary providers of its use.^63^ It is appropriate to discuss such issues with patients in an open and non-threatening conversation. Hot water bathing behaviors were once considered to be pathognomonic for CHS, but such behaviors have been documented in nearly half of CVS patients without cannabis use. The duration of cannabis cessation needed to confirm a CHS diagnosis is a point of contention. The accepted Rome Foundation criteria for CHS are qualitative in nature and do not provide any quantitative definition of duration and amounts of cannabis used or on how long a person needs to abstain from its use to be considered to have the disorder. Recent studies provide insight to address these limitations. In one analysis, only 19% of patients diagnosed with CHS abstained from cannabis for >4 weeks—far too short a time to ascribe cannabis use as causative of this episodic disorder. Also, Δ^9^-tetraydrocannabinol (THC) metabolites like THC-COOH are stored in adipose stores in chronic cannabis users and can remain in the system for ~3 weeks.^16^ Cannabis use disorder (CUD) includes nine patterns relating to impaired control and social function, risky behaviors, or evidence of physiological adaptation and is prevalent in CHS but not CVS.^1,64^ There is no clear relation between CHS and migraines or hormonal fluctuations as with CVS. CHS prevalence has risen in parallel with increasingly potent cannabis products that are higher in THC content.

Accurate characterization of CHS epidemiology has been challenging as diagnostic criteria are not universally adopted, and the Rome criteria for CHS are not practical given frequent reluctance of patients to abstain from cannabis for prolonged periods of time. CHS prevalence was calculated at 0.12% on a Rome survey study, but another group defining CHS as vomiting associated with cannabis use ≥20 days monthly and with symptom relief by hot showers calculated >10-fold higher prevalence rates.^65^

CHS pathogenesis is poorly understood. Chronic cannabis use is associated with downregulation of cannabinoid receptors and may lower emetic thresholds.^66^ Prolonged cannabis intake may cause nausea by desensitizing transient receptor potential vanilloid subtype 1 (TRPV1) receptors.^67^ Chronic cannabis users exhibit higher basal cortisol and ACTH levels, suggesting increased hypothalamic–pituitary axis activation and heightened stress response.^68^ THC and dronabinol delay gastric emptying but a role for gastric dysmotility in CHS is speculative.^69^ Gene polymorphisms of hepatic cannabinoid metabolizing enzymes including catechol-O-methyl transferase and CYP2C9 and polymorphisms of TRPV1, D_2_ receptor, and ATP-binding cassette transporter genes may lead to cannabinoid metabolite accumulation.^66,70^

Prolonged cannabis abstinence is the only accepted approach to resolve CHS episodes. CHS patients are believed to respond poorly to tricyclic prophylaxis, but some studies report improvements on these agents. A small CHS series noted reductions in emetic episodes on prophylaxis with olanzapine, a different neuromodulator.^71^ Abortive therapies reportedly effective in some CHS cases include D_2_ antagonists (haloperidol and droperidol), benzodiazepines, aprepitant, and topical capsaicin cream which may act in a similar fashion to hot showers to activate TRPV1 pathways. A systematic review of five observational studies and two randomized controlled trials noted mixed evidence of efficacy for capsaicin but positive clinical benefit from haloperidol and droperidol versus usual care or no comparator.^72^ In contrast, traditional antiemetics like ondansetron and metoclopramide appear to have limited efficacy in CHS.

Several topics are urgent priorities for further study in CHS. CHS diagnostic criteria must be refined based on available data and widely adopted in all clinical settings to better distinguish CHS from CVS. One proposal for updating criteria for CHS includes new quantitative cutoffs for duration of cannabis use prior to CHS onset (>1 year), amounts of cannabis use (≥4 days/week), and duration of cannabis abstinence to confirm CHS (>6 months or >3 typical emetic cycles).^16^ Cannabis cessation is the most challenging criterion to satisfy to confidently make a diagnosis of CHS. In one CHS study, cannabis use decreased transiently after ED visits but returned to pre-visit patterns within days.^73^ It has been proposed that patients unable to sustain abstinence be considered as having “suspected CHS” or “cannabis-related cyclic/episodic hyperemesis but without confirmed CHS”.^74^ Both clinical features and epidemiology warrant additional study in larger, multicenter cohorts. Defining CHS pathogenesis will help identify which cannabis properties are responsible for symptoms and which patients are predisposed to develop the condition. There are several goals of future treatment trials in CHS including determination if therapies for CVS truly are ineffective in CHS, if any neuromodulator agent exhibits prophylactic benefits in CHS as is seen with CVS (especially in those unable to eliminate cannabis), which non-traditional and traditional antiemetics help abort acute CHS attacks, and if there are pharmaceutical or psychological interventions to aid cannabis-dependent CHS patients abstain from further use. As with CVS, these ambitious aims can only be achieved with funding and generation of multicenter registries to support broad-based investigations using standardized inclusion criteria and study designs.

## 8 | CONCLUSIONS

Relevant information regarding the clinical presentations, pathogenesis, therapy, and socioeconomic impact of CVS and CHS has been published in recent decades which has improved our abilities to manage these challenging conditions. As detailed in the preceding sections, most findings have been obtained from small, single center series which have often been retrospective in nature. A unifying theme of each section of this article is the need to construct an adequately funded, robust multicenter infrastructure with standardized entry criteria and comprehensive validated survey instruments that will permit conduct of longitudinal natural history studies or controlled treatment trials.

## Figures and Tables

**TABLE 1:** Priorities for future investigation in cyclic vomiting syndrome.

Category	Investigative priorities
Clinical features	• Generate large databases to define breadth of clinical presentations and facilitate CVS subgroup analyses • Contrast clinical presentations in adults versus children to gain insight into age-related differences • Rigorously assess comorbidities to identify other potential targets for therapy • Identify triggers of CVS episodes to provide possible counseling to patients to avoid episodes • Characterize factors that accelerate resolution of episodes to be used in designing therapy protocols
Epidemiology	• Construct detailed international databases to identify CVS subsets based on age, ethnicity, geographic region (including outside US), and other factors • Perform initial analyses using claims data from payers • Recruit participation from industry and federal sources to complement claims data and to expand observations by including patients not covered by these payers
Pathophysiology	• Encourage involvement of prominent laboratories with expertise in basic and translational science • Focus on underlying neurologic pathways including migraines, other episodic conditions, and dysautonomia • Examine abnormalities in different neurotransmitter pathways including those relating to endocannabinoid and the hypothalamic–pituitary–adrenal axis • Continue detailed scrutiny of genetic factors relating to CVS • Examine impact of psychological dysfunction • In large, multicenter registries, determine interactions between the above proposed pathogenic factors
Treatment	• Devise and validate Patient-Reported Outcome instruments for consistent adoption in clinical trials in CVS • Conduct multicenter controlled trials of: • Novel abortive and prophylactic therapies (e.g., NK_1_ antagonists and CGRP antagonists) • Prophylaxis versus abortive therapies • Enroll patients in large multicenter registry to permit large sample recruitment that will allow subgroup analyses to determine selectivity of response to different therapies • Rigorously explore non-medication therapies such as transcutaneous auricular vagal stimulation, including comparison of different stimulation parameters • Focus on psychological treatment options

Abbreviations: CGRP, calcitonin gene-related peptide; NK_1_, neurokinin receptor subtype 1; TRPV1, transient receptor potential vanilloid subtype 1.

## Key points

1. Despite increasing understanding, there remain substantial gaps in knowledge about the clinical features, epidemiology, and management of CVS.
2. The pathophysiological basis for CVS and CHS is not well understood, and this lack of understanding is a barrier to developing new therapeutic approaches.
3. Increased investment from private and governmental sources is necessary to advance the rigor and scope of CVS research.
